# Modulation of bile acid profile by gut microbiota in chronic hepatitis B

**DOI:** 10.1111/jcmm.14951

**Published:** 2020-01-10

**Authors:** Xiaolin Wang, Lu Chen, Hui Wang, Wei Cai, Qing Xie

**Affiliations:** ^1^ Department of Infectious Diseases Ruijin Hospital Shanghai Jiao Tong University School of Medicine Shanghai China

**Keywords:** bile acids, chronic hepatitis B, fibrosis, gut microbiota

## Abstract

Chronic hepatitis B (CHB) is a global epidemic disease that may progress to fibrosis, cirrhosis and hepatocellular carcinoma. The role of the liver‐bile acid‐microbiota axis in CHB remains unclear. The aims of this study are to elucidate the alteration of the gut microbiota and its functions in bile acid homeostasis in CHB patients with different degrees of fibrosis. In the present study, we evaluated serum and faecal bile acid profiles in healthy controls and CHB patients with biopsy‐proven diagnosis: patients had stage 0‐1 fibrosis were classified as mild CHB and patients had stage 2‐4 fibrosis were classified as moderate/advanced CHB. The levels of serum total bile acids (BAs) and primary BAs were increased in CHB patients with moderate/advanced fibrosis, whereas faecal total and secondary BAs levels were significantly lower. Analyses of gut microbiota exhibited a trend of decreased abundance in bacteria genera responsible for BA metabolism in CHB patients with moderate/advanced fibrosis. CHB is associated with altered bile acid pool which is linked with the dysregulated gut microbiota. The higher level of FGF‐19 may act in a negative feedback loop for maintaining the bile acid homeostasis.

## 1 INTRODUCTION

Chronic hepatitis B (CHB) is a major public health problem that may progress to liver fibrosis, cirrhosis and hepatocellular carcinoma.^1^ The systematic monitoring of CHB patients and delaying disease progression to severe liver cirrhosis is urgently needed. Significant fibrosis is the hallmark of progression of CHB. To understand the mechanisms of progression of fibrosis is essential to prevention and individualize therapy in patients with CHB.

Increasing evidences suggested that bile acids (BAs) play a critical role in chronic liver diseases.^2^, ^3^ The alteration of BAs has been shown to be closely related with various liver diseases, like nonalcoholic fatty liver disease (NAFLD), alcoholic liver disease and cirrhosis and liver cancer.^4^, ^5^, ^6^, ^7^, ^8^ Studies on patients with NAFLD showed that the concentrations of serum primary and secondary BAs were increased and faecal secondary BAs production was elevated in patients with NAFLD.^4^ According to Genta et al, cirrhotic patients have been shown to have a higher serum primary BAs and the proportions of secondary BAs in bile were lower in advanced cirrhosis.^6^ A study performed by Wang et al showed that serum BAs are related with CHB‐induced cirrhosis.^9^

Bile acid metabolism is increasingly recognized as a critical player during liver‐microbiota crosstalk.^2^, ^10^ Gut microbiome convert primary BAs into secondary BAs and therefore possess potent effects on bile acid signalling. In addition, BAs are involved in the regulation of hepatic metabolism and inflammation, and also BAs synthesis via nuclear receptors, such as the farnesoid X receptor (FXR).^10^, ^11^, ^12^ Since BAs play an important role in pathophysiology, a better understanding of the relationship between BAs and gut microbiota will shed light on the pathophysiology of fibrosis.

In the present study, we enrolled treatment‐naïve CHB patients who underwent liver biopsy for the accurate diagnosis of liver inflammation and fibrosis. Given that fibrosis is progressively developed, it is an important characteristic that represents CHB progression. Thus, CHB patients were separated into two groups. Stage 0‐1 fibrosis was classified as mild CHB, and patients had stage 2‐4 fibrosis were classified as moderate/advanced CHB. We evaluated the gut microbiota composition and bile acid profiles in both the serum and faeces in CHB patients of each group. Our research revealed that CHB, especially CHB with moderate/advanced fibrosis, is related with a reduced conversion of faecal primary to secondary BAs which might be associated with the dysregulated gut microbiota.

## 2 MATERIALS AND METHODS

### 2.1 Participants

The criteria for diagnosing for CHB are according to the AASLD 2018 hepatitis B guidance.^13^ We enrolled treatment‐naïve CHB patients who underwent liver biopsy for the accurate diagnosis of liver inflammation and fibrosis. Patients with CHB were divided into two subgroups according to their fibrosis stages. Biopsy was used as the ‘gold standard’ for fibrosis diagnosis for all CHB patients. METAVIR scoring system was used for histopathology scores.

For the health control (HC) group, we recruited 21 healthy volunteers who visited the Ruijin Hospital for their annual physical examination. Exclusion criteria for the control group included diabetes, obesity, hypertension, NAFLD, chronic hepatitis, metabolic syndrome, IBD, coeliac disease and cancer. All participants who received probiotics and/or antibiotics within 8 weeks before the enrolment were also excluded.

This study has been approved by the Institutional ethics Review Committee at Ruijin hospital and was performed in compliance with the Declaration of Helsinki. Informed consents were obtained from all participants prior to participation.

### 2.2 Faecal microbiota analysis

Sample collection: Each participant provided a fresh stool sample that was delivered immediately to the laboratory in an ice bag. It was then divided into five aliquots of 200 mg and immediately stored at −80°C in the laboratory.

DNA extraction: Extraction of DNA from stool was performed following the protocol of the QIAamp DNA stool minikit (Qiagen).

PCR amplification and sequencing: PCR amplification and sequencing were conducted at Realbio Genomics Institute (Shanghai, China). The V3‐V4 region of the bacteria 16S ribosomal RNA genes was amplified by PCR using primers 341F 5′‐CCTACGGGRSGCAGCAG)‐3′ and 806R 5′‐GGACTACVVGGGTATCTAATC‐3′. Amplicons were extracted from 2% agarose gels and purified using the AxyPrep DNA Gel Extraction Kit (Axygen Biosciences) according to the manufacturer's instructions and quantified using Qubit®2.0 (Invitrogen). After preparation of library, these tags were sequenced on MiSeq platform (Illumina, Inc) for paired end reads of 250 bp, which were overlapped on their three ends for concatenation into original longer tags.

Process of sequencing data: Tags, trimmed of barcodes and primers, were further checked on their rest lengths and average base quality. 16S tags were restricted between 220 and 500 bp such that the average Phred score of bases was no worse than 20 (Q20) and no more than three ambiguous N. Operational taxonomic units (OTUs) were clustered with 97% similarity using UPARSE (http://drive5.com/uparse/), and chimeric sequences were identified and removed using Userach (version 7.0). Each representative tags was assigned to a taxa by RDP Classifier (http://rdp.cme.msu.edu/) against the RDP database (http://rdp.cme.msu.edu/) using confidence threshold of 0.8. OTU profiling table, and alpha/beta diversity analyses were also achieved by python scripts of Qiime.

### 2.3 Measurement of BAs

Bile acids analysis was performed on the UPLC‐MS/MS system (ACQUITY UPLC‐Xevo TQ‐S, Waters Corp.) according to previously published methods.^14^ The raw data files generated by UPLC‐MS/MS were processed using QuanMET software (v1.0, Metabo‐Profile) to perform calibration and quantitation for each bile acid.

### 2.4 FGF19 ELISA

Serum FGF19 measurement was performed using an ELISA kit from R&D Systems following the manufacturer's protocol.

### 2.5 Statistical analysis

A comparison of two groups was conducted by unpaired Student's *t* test for continuous variables and chi‐square test for categorical variables. And comparisons of data of 16sRNA or BA assay were conducted by using nonparametric statistics. The correlations were evaluated by Spearman's correlation analysis.

Statistical analyses were conducted by SAS version 9.3; Graphs were done using GraphPad prism 7.0.

## 3 RESULTS

### 3.1 Clinical characteristics of the recruited CHB patients and healthy individuals

This study included 69 CHB patients with biopsy‐proven fibrosis stages: 25 patients had stage 0‐1 fibrosis and were classified as mild CHB (Group G1) and 44 patients had stage 2‐4 fibrosis and were classified as moderate/advanced CHB (Group G2). Table 1 provides a detailed demographic, biochemical and clinical profile of the entire cohort. Patients with moderate/advanced fibrosis were more likely to have higher TB, DB, HBV DNA and liver stiffness than those without moderate/advanced fibrosis.

**Table 1:** Clinical information of human subjects

	HC (n = 21)	CHB (n = 69)	*P* value#	Fibrosis Stage 0‐1, G1 (n = 25)	Fibrosis Stage 2‐4, G2 (n = 44)	*P* value*
Age (y)	38.29 ± 8.95	39.09 ± 10.13	0.745	37.24 ± 59.09	40.14 ± 10.63	0.172
Male (number)	16 (76%)	53 (78%)	0.545	16 (64%)	37 (84%)	0.100
BMI (kg/m^2^)	23.78 ± 2.38	23.31 ± 2.89	0.500	23.16 ± 3.57	23.47 ± 2.58	0.749
ALT (IU/L)	23.29 ± 12.85	47.34 ± 32.56	0.059	36.26 ± 21.00	48.05 ± 35.11	0.079
AST (IU/L)	18.43 ± 10.05	36.40 ± 20.69	0.029**#**	32.00 ± 9.20	38.79 ± 24.04	0.272
ALP (IU/L)	55.86 ± 16.26	70.67 ± 13.64	0.011**#**	65.73 ± 14.87	73.03 ± 12.84	0.077
GGT (IU/L)	25.00 ± 11.87	34.25 ± 25.76	0.357	25.67 ± 15.06	38.09 ± 29.09	0.083
TB (μmol/L)	12.86 ± 6.28	16.53 ± 7.45	0.218	13.67 ± 7.00	17.75 ± 7.47	0.024*
DB (μmol/L)	2.26 ± 0.60	3.07 ± 1.63	0.207	2.40 ± 1.23	3.32 ± 1.74	0.036*
HBsAg (IU/mL)	negative	16 103.06 ± 26 750.83		21 049.10 ± 28 682.25	14 978.29 ± 26 765.95	0.142
HBeAg^+^ n (%)	0(0.00%)	26(37.68%)	0.001#	10(40.00%)	16(36.36%)	0.090
Lg HBV DNA (IU/mL)	negative	5.10 ± 2.02		4.88 ± 2.40	5.17 ± 1.80	0.045*
Liver stiffness (Kpa)				6.65 ± 1.778	8.86 ± 5.89	0.021*
CAP (dB/m)				212.07 ± 62.17	204.07 ± 46.09	0.125

Note

Date are presented as mean ± SD. Comparison between two groups was performed using Mann‐Whitney *U* test or chi‐square test as appropriate.

Abbreviations: ALP, alkaline phosphatase; ALT, alanine aminotransferase; AST, aspartate aminotransferase; BMI, body mass index; CAP, controlled attenuation parameter; DB, direct bilirubin; GGT, gamma glutamyl transpeptidase; HC, health control; TB, total bilirubin.

^#^ *P* value comparison between HC and CHB group.

* *P* value comparison between G1 and G2 group.

### 3.2 Serum bile acid levels and bile acid composition

**Figure 1:**
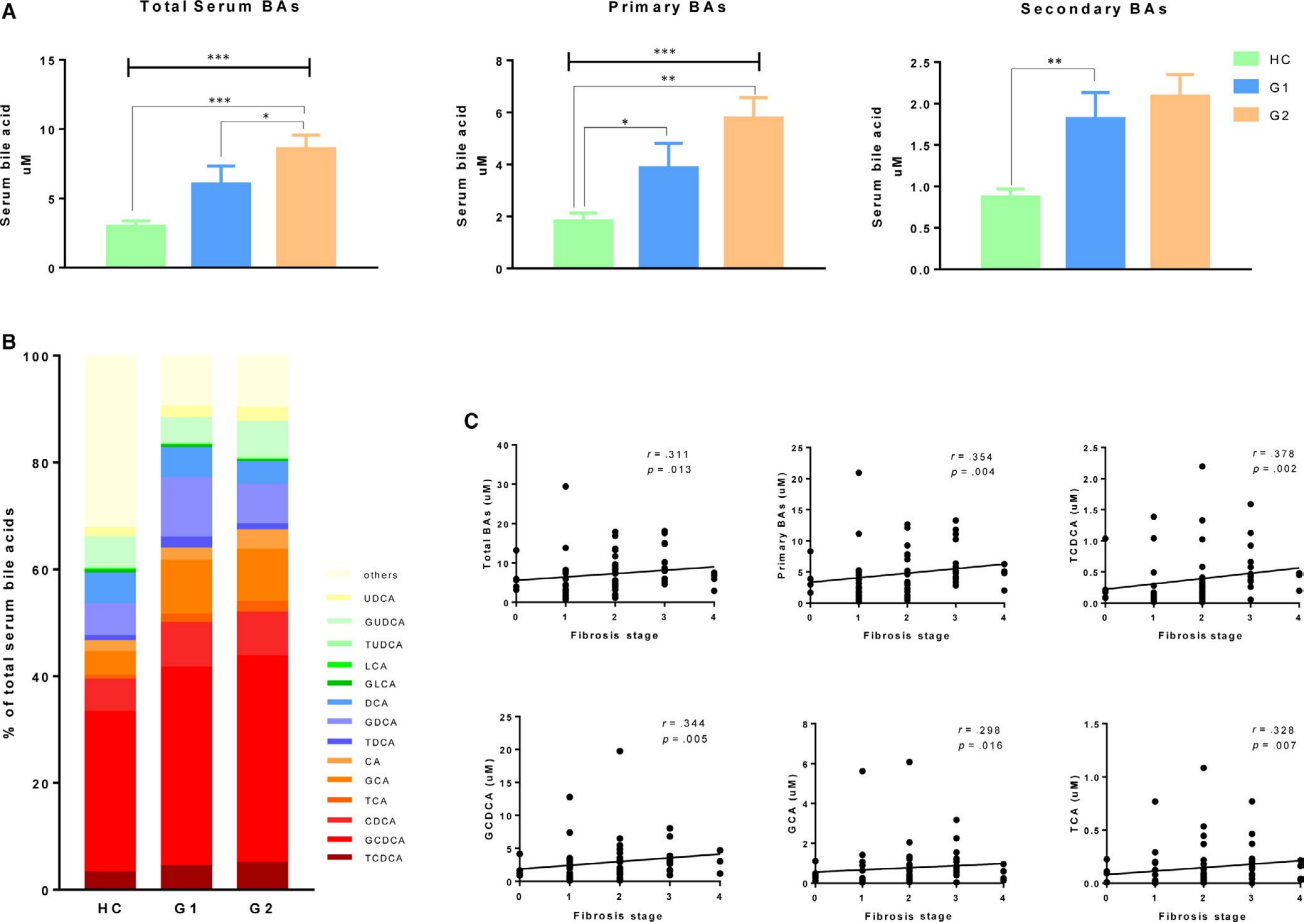
Serum bile acids profiles in patients with chronic hepatitis B. A, Total bile acids and primary and secondary levels of serum bile acids. B, Serum bile acid composition (% of total serum bile acids). C, The correlations between the fibrosis stage and total serum BAs, total primary BAs, TCDCA, GCDCA, GCA and TCA

We first studied the serum BA profiles in CHB patients with different liver fibrosis stages. As shown in Figure 1A, total BAs and total primary BAs showed a stepwise increase across the three groups. Levels of secondary BAs also showed a trend of increase but with no significance. Due to the higher total BA levels in CHB patients, we then studied the percentage of each BA (BA concentration/total BAs concentration) among the three groups. The relative proportion of BAs was shown in a stacked bar plot in Figure 1B, comparing the amount of BAs in HC and in patients in G1 group and in G2 group. CHB patients with moderate/advanced fibrosis had the highest relative proportion of total primary BAs. We further analysed the correlations between BAs and liver fibrosis stage. As shown in Figure 1C, significantly positive relationships were observed between the fibrosis stage and total serum BAs, total primary BAs, TCDCA, GCDCA, GCA and TCA. These results indicated an increase in serum BAs, especially conjugated primary BAs in CHB patients with moderate/advanced fibrosis.

### 3.3 Faecal bile acid levels and bile acid composition

**Figure 2:**
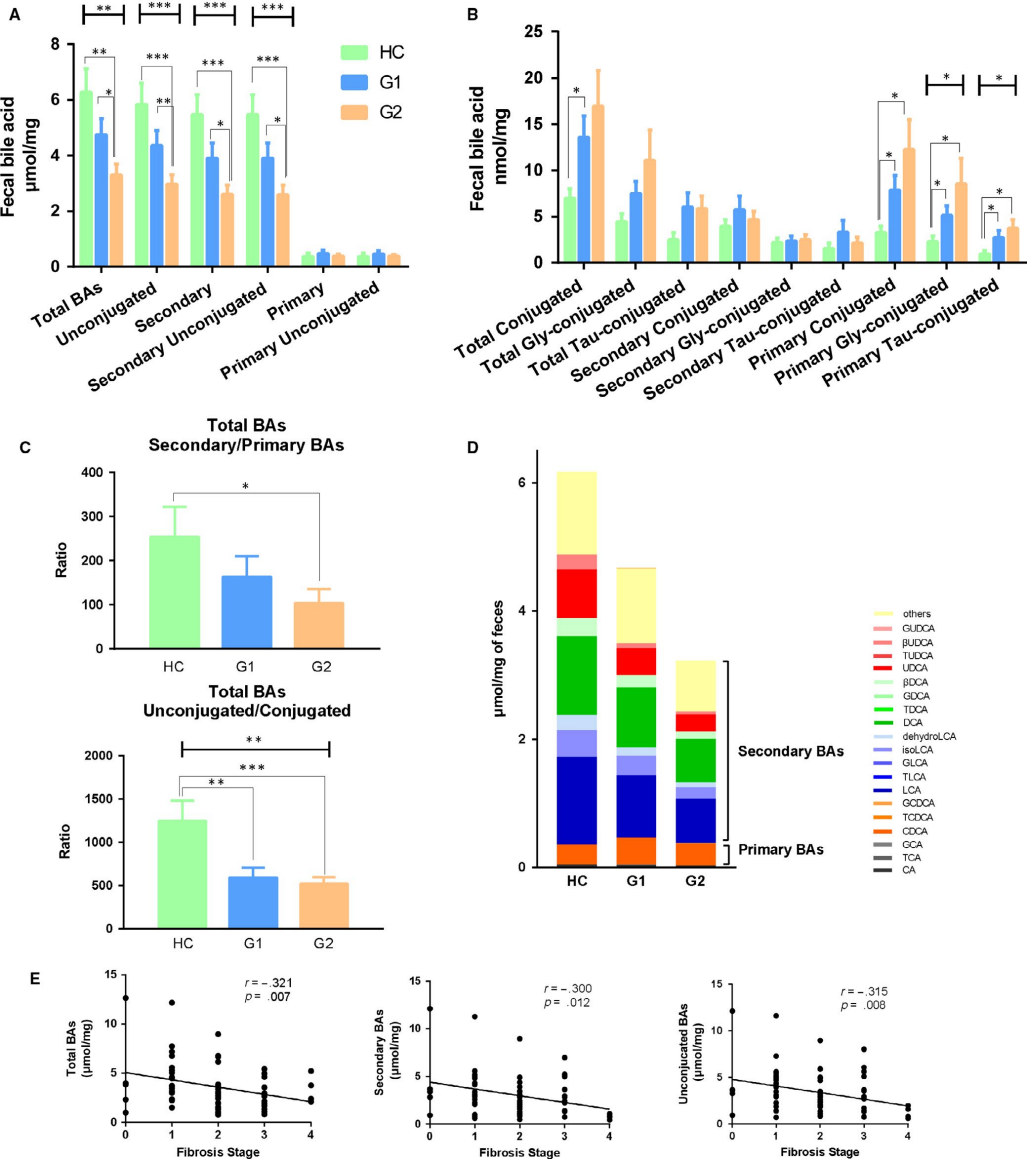
Faecal bile acid profiles in patients with chronic hepatitis B. A, Total bile acids and secondary, primary and unconjugated bile acids levels. B, The conjugated bile acid levels. C, The ratio of secondary/primary BAs and unconjugated/conjugated BAs. D, Faecal bile acids composition of faecal bile acids. E, The correlations between the fibrosis stage and total faecal BAs, total secondary BAs and unconjugated BAs

We further studied the faecal BAs profile in HC and CHB patients. As shown in Figure 2A, the total faecal BAs, unconjugated BAs, total faecal secondary BAs and unconjugated secondary BAs were decreased in a stepwise manner from control to G1 to G2 group. Moreover, a stepwise decrease in secondary BAs, LCA, DCA, isoLCA, 6_keto LCA, 7_keto LCA, 12_keto LCA, dehydro LCA and 3βDCA (3β‐deoxycholic acid) were observed from control to G1 to G2 group (Figure S1B). Conversely, the total primary BAs and unconjugated primary BAs were not different across the study groups, while a significant trend for increased conjugated primary BAs was noted (Figure 2B and Figure S1A).

Interestingly, we found a stepwise decrease in the ratio of secondary to primary BAs and also the ratio of unconjugated to conjugated BAs across the three groups (Figure 2C). The amount of primary and secondary BAs was showed in a stacked bar plot in Figure 2D. Faecal total BAs and secondary BAs were obviously decreased in a stepwise manner from control to G1 to G2 group. We further studied the correlation between the BAs profiles with fibrosis stage. A higher grade of fibrosis was associated with a significantly decreased total faecal BAs, total secondary BAs and total unconjugated BAs in a linear fashion as shown in Figure 2E.

### 3.4 Gut microbiota profiles

**Figure 3:**
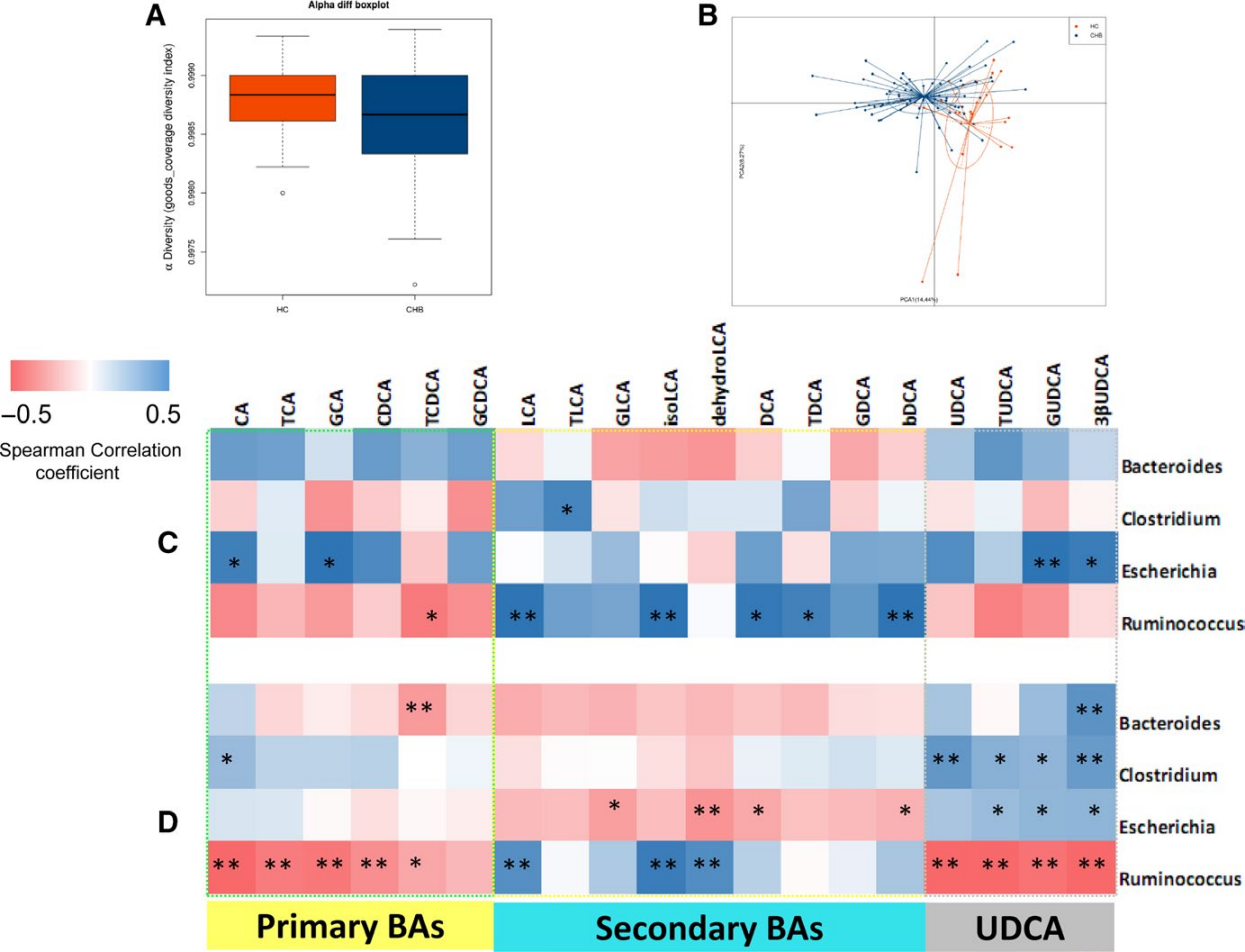
Gut microbiota profiles in CHB patients. A and B, Alpha diversity and principal coordinates analysis (PCA) for the weighted UniFrac distance of the gut microbiota of hepatitis B (CHB) patients (blue) and controls (orange). C and D, Heatmap representation of the Spearman's r correlation coefficient between bacterial taxa (genus level) and bile acids profiles in faecal of control group (C) and CHB group (D)

Intestinal bacteria were known to regulate bile acid metabolism through biotransformation.^11^ The dysregulated BAs identified in CHB patients prompted us to examine the gut microbiota associated with bile acid metabolism. As shown in Figure 3A, the alpha diversity of gut microbiota in CHB patients was lower than that of healthy subjects. The PCA plot showed a shift in the overall gut microbiota composition from CHB patients to healthy subjects (Figure3B). We further assessed the correlations between BAs with the bacteria species. Faecal primary BAs were mostly positively correlated with Escherichia in HC, while in CHB patients, faecal primary BAs were negatively correlated with Ruminococcus. Secondary BAs levels were mostly positively correlated with Ruminococcus in HC, while negatively correlated with Escherichia in CHB patients. GUDCA (glycoursodeoxycholic acid) and 3βUDCA (3β‐ursodeoxycholic acid) were positively correlated with Escherichia in HC, while most UDCAs were positively associated with Bacteriodes, Clostridium and Escherichia and negatively associated with Ruminococcus in CHB patients (Figure 3C and 3D). These correlations suggest that the interaction between bacterial genera involved in BA metabolism and intestinal BAs profiles were changed in CHB patients.

**Figure 4:**
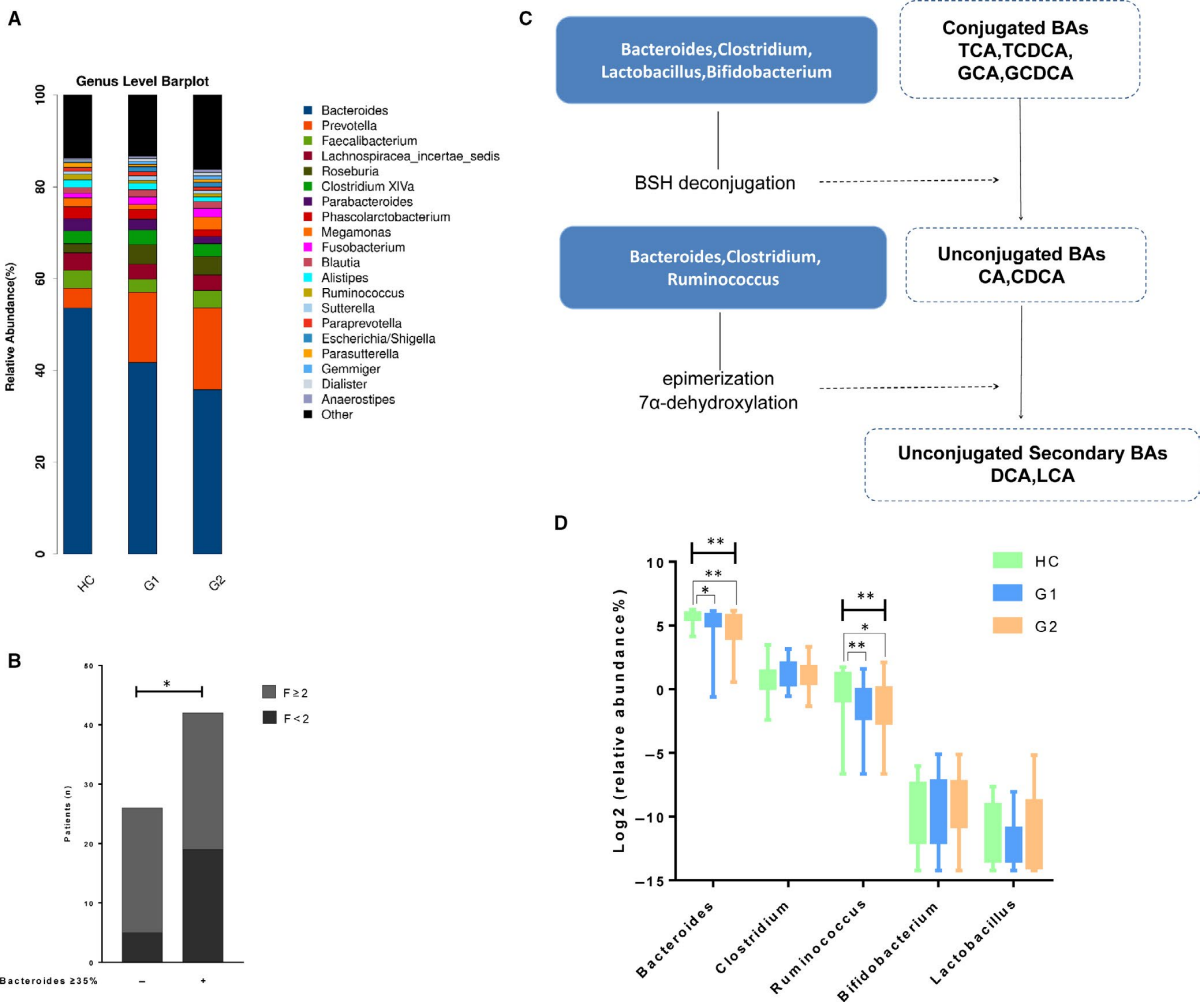
Altered gut microbiomes in HC and in CHB patients with different fibrosis stages. A, Taxonomic composition of the gut microbiota in each group. B, Number of patients with fibrosis F ≥ 2 and F < 2 according to Bacteroides abundance. C, Schematic depicting the roles of intestinal bacteria in BA metabolism. D, The abundances of BA‐biotransforming bacteria in the faeces of patients with CHB and healthy controls

When comparing the gut microbiota in HC and in CHB patients with different fibrosis stages, significant differences appear at the genus level (Figure 4A): Bacteroides decreased in CHB, especially in G2 group, whereas Prevotella increased. As previously described,^15^ these two genera usually showed an inverse relationship in their abundance between each other. As Bacteroides were significantly decreased in G2 group, we evaluated the severity of fibrosis in CHB patients according to two subgroups defined by the abundance of Bacteroides < 35% and Bacteroides ≥ 35%. The rate of fibrosis stage F ≥ 2 was, respectively: 80.77%, and 54.76% (*P* = 0.02, Figure 4B). Considering that Bacteroides belongs to the main bacterial genera involved in BA metabolism, we further studied the bacterial taxa that responsible for the key steps of bile acid conversion. Primary bile acids were mostly deconjugated via the action of bile salt hydrolase (BSH).The bacteria Bacteroides, Clostridium, Lactobacillus and Bifidobacterium belongs to the main bacterial genera that express BSH.^11^, ^16^, ^17^ Bacteria capable of epimerization include Bacteroides, Clostridium and Ruminococcus.^11^, ^18^ 7‐alpha‐dehydroxylation is mainly performed by Clostridium.^19^ As illustrated in Figure 4D, the abundances of Bacteroides and Ruminococcus were significantly lower in CHB patients than healthy controls. The above data suggest that gut dysbiosis is correlated with CHB fibrosis progression, possibly via bile acid metabolism.

### 3.5 Increased serum FGF19 in patients with CHB

**Figure 5:**
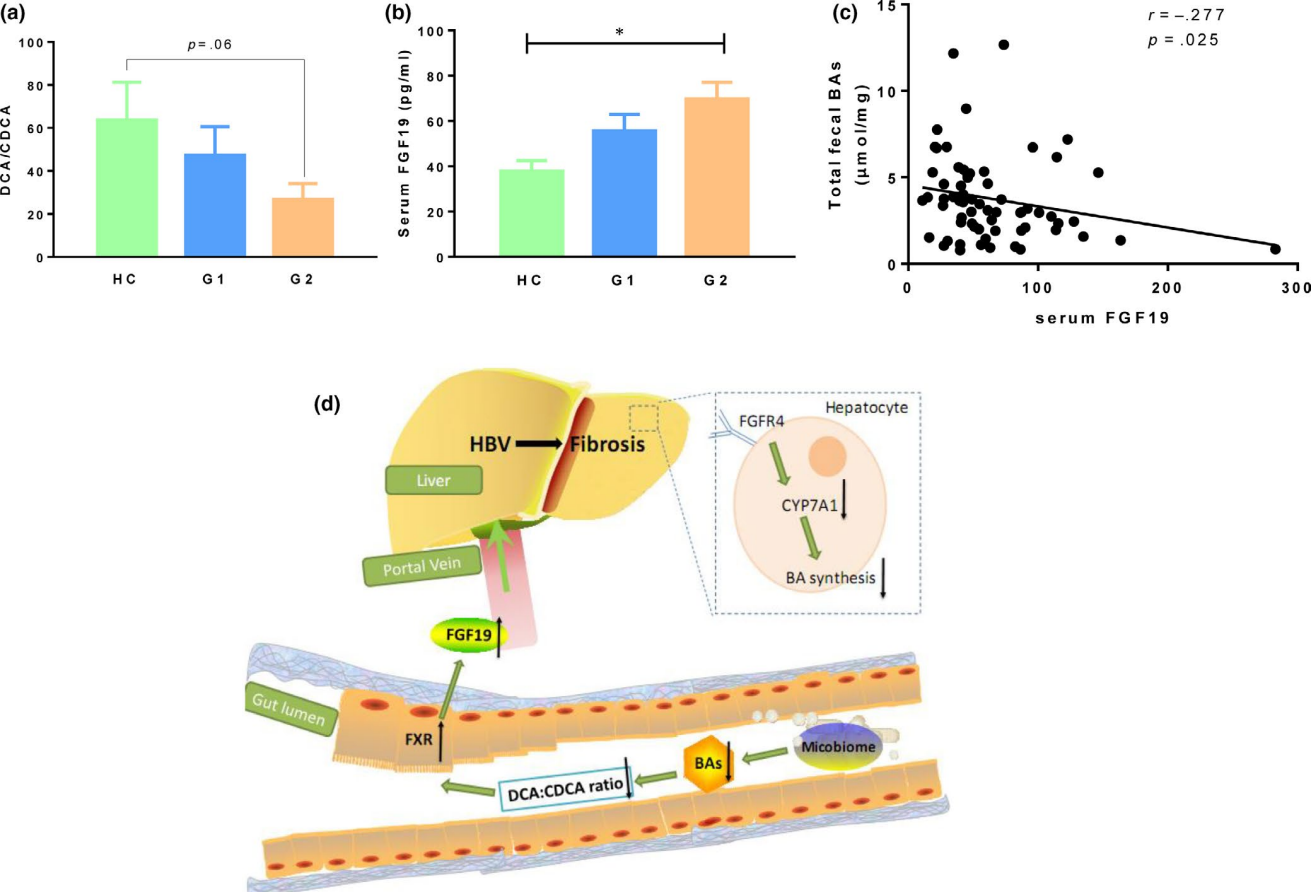
Gut microbiota have an effect of negative feedback on bile acid metabolism. A, The ratio of faecal DCA to CDCA in control and CHB patients. B, Serum FGF19 in control and CHB patients. C, The correlation between total faecal BAs and serum FGF19 level. D, Diagrammatic representation of liver‐bile acid‐microbiota axis. The gut microbiota are altered in CHB induced fibrosis, reducing the conversion of primary to secondary bile which in turn lead to increase the activation of intestinal FXR. Elevated FGF19 which enters portal circulation and binds to the FGFR4, downregulating CYP7A1 within hepatocytes, further reduces the production of primary bile acids by the liver

Intestinal BAs bind to FXR, producing fibroblast growth factor 19 (FGF19), which reaches the liver via portal circulation. FGF19 then binds to fibroblast growth factor receptor 4 (FGFR4), resulting in the downregulation of BAs synthesis.^12^, ^20^ BAs differ in their potentials to activate FXR.^21^ A higher ratio of DCA: CDCA was considered to reduce the activation of intestinal FXR, leading to lower expression of FGF19.^21^, ^22^, ^23^ We further examined the ratio of faecal DCA to CDCA. As shown in Figure 5A, the ratio of DCA to CDCA showed a decreased trend toward the three groups, indicating that the activation of intestinal FXR by faecal BAs was elevated in CHB patients with moderate/advanced fibrosis. Besides, we observed a stepwise increase in serum FGF19 from control to G1 to G2 group (Figure 5B). In line with these results, the serum FGF19 level showed a negative relationship with the concentration of total faecal BAs (Figure 5C). We speculated that elevated FGF19 inhibits the synthesis of primary BAs by the liver (Figure 5D).

## 4 DISCUSSION

Significant fibrosis is an important disease driver in CHB. However, the role of liver‐bile acid‐microbiota axis in the progression of CHB towards fibrosis is unclear. In the current study, we provided several findings illustrating that the ability of gut microbiota to convert primary to secondary bile acids is reduced in chronic hepatitis B patients with moderate/advanced fibrosis.

Developing researches have demonstrated the roles of gut microbiota in liver diseases.^24^, ^25^ The correlations between gut microbiome and HBV‐induced chronic liver diseases have been recently studied.^26^ Lu et al showed that the B/E ratio (Bifidobacteriaceae/Enterobacteriaceae) was significantly decreased in CHB patients, especially in patients with cirrhosis.^27^ In the present study however, the B/E ratio did not show significant difference between the predefined subgroups (data not show). Interestingly, we found that bacteria that involved in BAs metabolism were decreased in CHB patients, suggesting that the altered intestinal microbiota might contribute to impaired bile acids circulation. Our study implied that gut microbiota could be at least one of the mechanisms that explain the altered bile acids pool in CHB progression.

Bile acids, synthesized in the liver, are metabolized by gut bacteria in intestine and are critical for maintaining the host metabolism.^24^, ^28^ The changes of bile acid profiles in different liver conditions are inconsistent.^4^, ^5^, ^6^, ^7^ Growing evidences suggested that cirrhosis is closely related with bile acid metabolism.^6^, ^9^ In this study, we observed the increased amount of BAs in the serum of CHB patients with the progression of fibrosis. This result was in accordance with Xiaoning Wang et al’s study, which showed the dynamic alteration of some bile acids among different stages of hepatitis B‐induced cirrhosis.^9^ In our current study, total serum BAs, total primary BAs, TCDCA, GCDCA, GCA and TCA showed significantly positive correlations with the fibrosis stages. The increase in the serum BAs can be partly explained by the cholestasis, which was also demonstrated by increased GGT and bilirubin levels in CHB patients with moderate/advanced fibrosis.

Bile acids in the intestinal bind to FXR, producing FGF19, which enters portal circulation and then reaches the liver.^29^ Binding of FGF19 to its receptor FGFR4 in liver results in the inhibition of BAs synthesis. The elevated serum FGF19 was previously showed in patients with liver diseases including alcoholic hepatitis, noncirrhotic and cirrhotic primary biliary cholangitis.^29^, ^30^ To further explore the signalling of intestinal BAs in CHB, we analysed the ratio of faecal DCA/CDCA, which represent the potentials of BAs in activation of intestinal FXR.^22^, ^23^ The decreased DCA/CDCA ratio is in accordance with elevated serum FGF19. In line with these results, the serum FGF19 level showed a negative relationship with total faecal BAs. The elevated FGF‐19 could inhibit the production of primary bile acids by the liver. However, the high levels of FGF‐19 are probably insufficient to counteract the increased serum bile acids pool due to the cholestasis. We speculated that the increased serum bile acids and the higher levels of FGF‐19 might act in a negative feedback loop by inhibiting bile acid synthesis for maintaining the systematic bile acid homeostasis. Further studies are needed to analyse the activity of intestinal FXR and also the BAs synthesis in CHB patients.

In conclusion, our current study revealed that CHB, especially CHB with moderate/advanced fibrosis, is correlated with a decreased conversion of primary to secondary BAs in the intestinal which is associated with the dysregulated gut microbiota. Based on these findings, we speculated that elevated FGF19 which binds to the FGFR4, downregulating CYP7A1 in hepatocytes, further reduces the synthesis of primary BAs in CHB patients. These changes in gut microbiota could be considered as a protective mechanism to decrease overall BAs of the body. Overall, modulation of gut microbiota or bile acid metabolism could represent a promising target for treatment of fibrosis in CHB patients.

## CONFLICTS OF INTEREST

We do not have any conflict of interests to be declared.

## AUTHOR CONTRIBUTIONS

XW designed and conducted the experiments and wrote the paper; XW and LC collected and prepared the serum and faecal samples; XW extracted DNA from stool; and HW, WC and QX supervised the whole project.

## Data Availability

The data that support the findings of this study are available on request from the corresponding author. The data are not publicly available due to privacy or ethical restrictions.
