# Supplementary figures and images for: Modulation of bile acid profile by gut microbiota in chronic hepatitis B
Source: J Cell Mol Med. 2020 Jan 10;24(4):2573–81. doi: 10.1111/jcmm.14951 (PMC7028859; doi:10.1111/jcmm.14951)

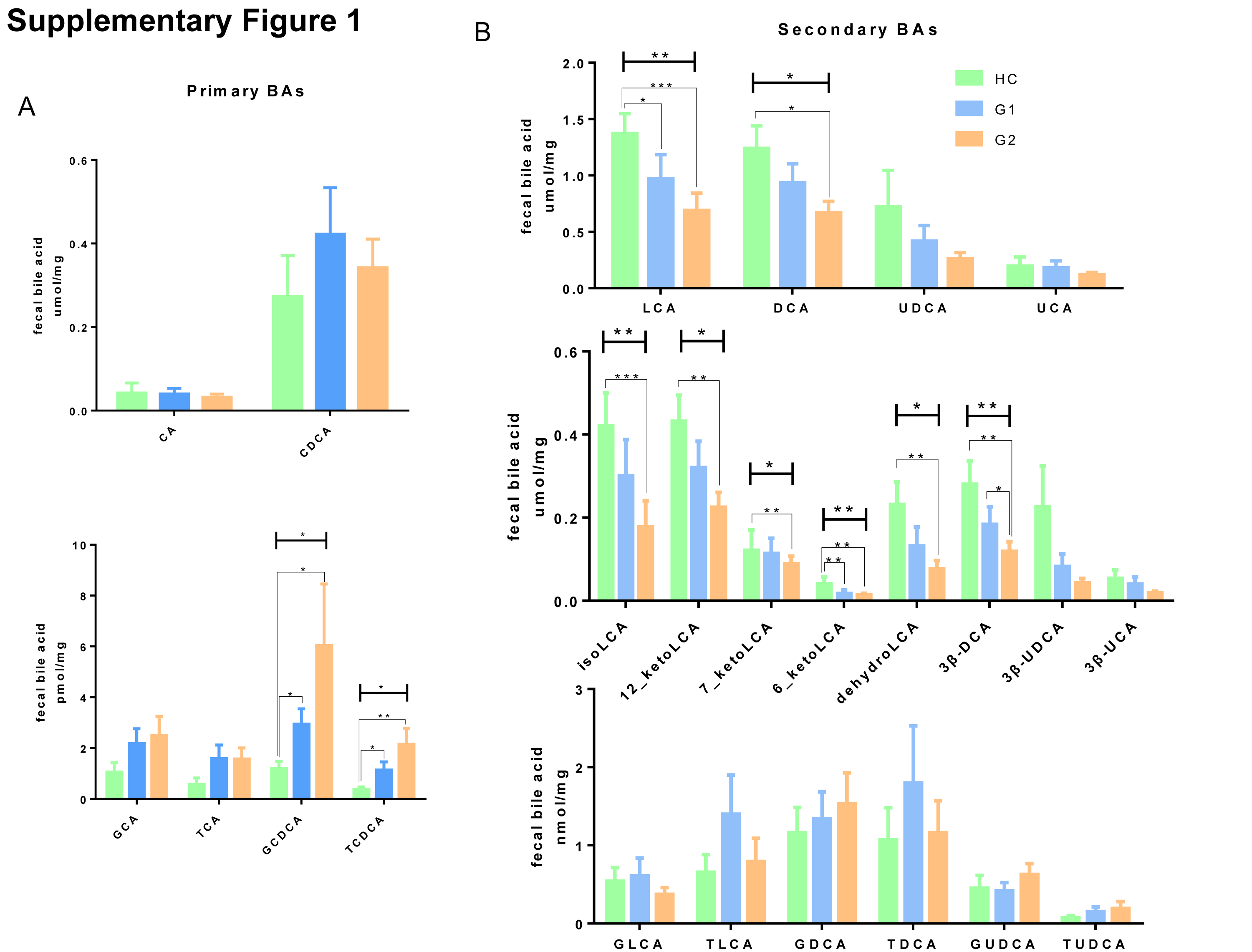

Supplement: Supplementary file 1 [file JCMM-24-2573-s001.tif]
