# Supplementary material for: Modulation of bile acid profile by gut microbiota in chronic hepatitis B
Source: J Cell Mol Med. 2020 Jan 10;24(4):2573–81. doi: 10.1111/jcmm.14951 (PMC7028859; doi:10.1111/jcmm.14951)
Supplement: Supplementary file 2 [file JCMM-24-2573-s002.docx]

**Supplementary Figure Legends**

Supplementary Figure 1. Distinct fecal primary (A) and secondary (B) BA changes in CHB.
